# Granulosa cell tumor in a six-year-old girl presented as precocious puberty

**Published:** 2010

**Authors:** Mahin Hashemipour, Mohammad Hassan Moaddab, Masoud Nazem, Parvin Mahzouni, Mehdi Salek

**Affiliations:** a Pediatric Endocrinologist, Isfahan Endocrine and Metabolism Research Center, Isfahan University of Medical Sciences, Isfahan, Iran; b Associate Professor of Pediatric Surgery, Department of Pediatric Surgery, School of Medicine, Al-Zahra Hospital, Isfahan University of Medical Sciences, Isfahan, Iran; c Associate Professor of Pediatric Surgery, Department of Pediatric Surgery, School of Medicine, Al-Zahra Hospital, Isfahan University of Medical Sciences, Isfahan, Iran; d Pediatrician, Isfahan Endocrine and Metabolism Research Center, Isfahan University of Medical Sciences, Isfahan, Iran

**Keywords:** Granulosa Cell Tumor, Puberty, Children

## Abstract

Ovarian sex cord-stromal tumors, including granulose cell tumors (GCTs), are a group of neoplasms that occur rarely, especially in children. Only 0.1 percent of all ovarian tumors and 4-5 percent of GCTs occur in children. The most common presentation of these tumors in children is precocious puberty. We report a 6 years old girl with isosexual precocity, presented as premature thelarche and vaginal bleeding. Ultrasonography of pelvis showed a hypoechoic solid mass of left ovary.

Microscopic features of the resected mass were characteristic of juvenile GCT. Although in most of girls with precocious puberty, the etiology is idiopathic, important causes, such as ovarian tumors must be considered.

Granulosa cell tumors of ovary first were described by Rokitansky in 1855.^1^ There are a group of cells in juvenile granulosa cell tumors (JGCT) with ability to secret steroid hormones, similar to the steroid hormone-secreting cells.^2^ They are benign tumors as a subgroup of ovarian sex cord-stromal tumors.

Pseudo precocious puberty is the most common presentation in young children. In older ages and adolescents, they cause other manifestations such as hirsutism, abnormal uterine bleeding and abdominal discomfort.

The prognosis of the JGCT is excellent after salpingooophorectomy in patients who have only ovarian involvement.^3^ This report describes a 6-year-old girl with isosexual precocity as premature thelarche and vaginal bleeding. Ultrasonography of pelvis showed a hypoechoic solid mass in close proximity of the left ovary. Microscopic features of the resected mass were characteristic of JGCT.

## Case Report

**Figure 1:**
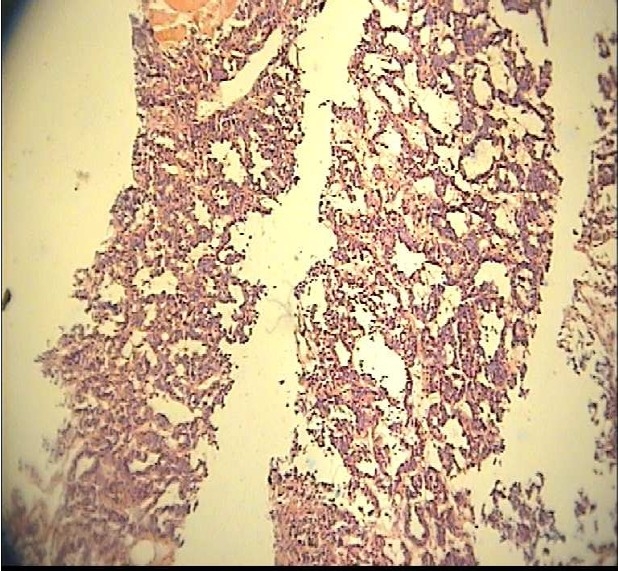
Microscopic specimen of juvenile granulose cell tumor of 6-year-old child with precocious puberty.

A girl, aged 6 years, with no significant past medical history presented with breast enlargement and intermittent periumblical abdominal pain of 4 months duration that was accompanied with nausea but without vomiting. She had no headache or visual complaint. At that time, medroxy progesterone, 5 mg PO daily was started with impression of pseudo-precocious puberty, because sonography of pelvis showed a cystic mass in left ovary. After several weeks, she developed intermittent vaginal bleeding and the drug was increased to 10 mg PO daily and LHRH agonist was added and she was referred to the Pediatric Endocrinology clinic in our medical center. When she was visited, her weight was 21 kg and her height was 120 cm. Breast enlargement and pubic hair were compatible with Tanner III. No abdominal mass was palpated in the abdomen. Her serum laboratory findings were consistent with peripheral precocious puberty: estradiol: 216 pmol/L; FSH: less than 0.5 IU/L; LH: less than 0.4 IU/L; DHEA-so4: less than 36 ng/ml; testosterone (total): 0.3 ng/ml; 17-OH-progesterone: 0.3 ng/ml; total T4: 9.1 μ g/dl; TSH: 2.4 mIU/L; alpha-fetoprotein: 2 ng/ml. LHRH test was done without increase in LH and FSH. Ultrasonography of pelvis showed a 6.2 × 5 × 4.1 cm hypoechoic solid mass in projection of left adnexa which had multiple cystic structures up to 1.2 cm, and left ovary could not be separated from it. Right ovary was normal. The size of uterus was appropriate for the patient’s age, with normal endometrial and myometrial echo pattern. dometrial thickness was 5 mm and no free pelvic fluid was detected by sonography. The spiral CT scan of pelvis showed a large non-uniform solid mass with multiple necrotic regions in the lower abdomen. So, we recommended diagnostic and therapeutic laparotomy for her. Under general anesthesia, laparotomy was done. An ovarian mass in the left side with diameters of 5 × 7 cm was detected and resected with left ovary. The right ovary was normal. No lymphadenopathy was detected in the pelvic area. The mass was sent for histopathologic study and ovarian JGCT was reported (Figure 1.)

## Discussion

Ovarian sex cord-stromal tumors are neoplasms containing granulosa cells, sertoli cells, theca cells, leydig cells, and fibroblasts of gonadal stromal origin.^4^ GCTS can be categorized into juvenile and adult subtypes based on their histology.^5^ Only 0.1 percent of all ovarian tumors and 4-5 percent of GCTs occur in children.^6^ Isosexual precocity occurs in 70-80 percent of prepubertal girls with GCTs.^6^–^8^ However, only 1 percent of all cases of sexual precocity in prepubertal girls are due to granulosa and theca cell tumors.^9^ Clinicians should be fully aware of pseudoprecocious puberty. There is high level of sex hormones and suppressed gonadotropins level in this condition.

They should exclude primary gonadal disorders, adrenal gland, exogenous secretion of gonadotropin by a tumor, or autonomous ovarian cyst.^10^ Patients with recurrent ovarian cyst formation, and persistence of the cysts especially with a significant solid component beyond three months should alert a clinician to the possibility of the ovarian JGCT.^10^ In our case, sonography of the pelvis showed ovarian cyst of solid component. An extensive review of the literature showed that primary ovarian tumors and steroid cell tumors are rarely seen in prepubertal children, less than 5%.^11^ In reviewing the literature from 1925 to 1944, seckel found 31 case reports of ovarian granulose cell tumors with sexual precocity.^12^ The signs of isosexual precocity are reported to develop uniformly. Alveolar pigmentation and breast development are the initial changes, followed by a white vaginal discharge and uterine bleeding, and finally, by development of downy pubic hair.^13^

Advanced bone age and accelerated height velocity were seen due to tumor-derived estradiol.^3^ Our patient was presented with breast enlargement followed by vaginal bleeding, while restlessness, irritability, and fever have been noticed in some patients,^14^ and increased sweating, apathy and ascitis are reported in others.^15^ About 90 percent of JGCT are diagnosed in stage 1 and have a good outcome, but those at progressive stages have poor prognosis.^11^ On CT and sonography, they most typically appear as large, multilocular masses, with either thin septations or thick, irregular septations, as well as solid components or large multicystic mass.^16^ In our patient, the sonography and CT scan of pelvis showed a solid ovarian mass. After confirmation of the diagnosis, appropriat treatment should be instituted. Surgery should be performed in this age group with unilateral oophorectomy only for stage one.^17^,^18^ Adjuvant chemotherapy is not useful for stage one.^19^ In our patient, left oo-phorectomy was done and the response to treatment was good after 4-6 months of follow up with regression in breast size and decrease in the serum estradiol level.

## References

[1] Calaminus G, Wessalowski R, Harms D, Gobel U (1997). Juvenile granulosa cell tumors of the ovary in children and adolescents: results from 33 patients registered in a prospective cooperative study. Gynecol Oncol.

[2] Harris AC, Wakely PE, Kaplowitz PB, Lovinger RD (1991). Steroid cell tumor of the ovary in a child. Arch Pathol Lab Med.

[3] Merras-Salmio L, Vettenranta K, Mottonen M, Heikinheimo M (2002). Ovarian granulosa cell tumors in childhood. Pediatr Hematol Oncol.

[4] Aboud E (1997). A review of granulosa cell tumours and thecomas of the ovary. Arch Gynecol Obstet.

[5] Lack EE, Perez-Atayde AR, Murthy AS, Goldstein DP, Crigler JF, Vawter GF (1981). Granulosa theca cell tumors in premenarchal girls: a clinical and pathologic study of ten cases. Cancer.

[6] Breen JL, Maxon WS (1997). Ovarian tumors in children and adolescents. Clin Obstet Gynecol.

[7] Boles ET, Hardacre JM, Newton WA (1961). Ovarian tumors and cysts in infants and children. AMA Arch Surg.

[8] Velasco-Oses A, Alonso-Alvaro A, Blanco-Pozo A, Nogales FF (1988). Ollier’s disease associated with ovarian juvenile granulosa cell tumor. Cancer.

[9] Pedowitz P, Felmus LB, Mackles A (1955). Precocious pseudopuberty due to ovarian tumors. Obstet Gynecol Surv.

[10] Low LCK, Wang Q (1998). Gonadotropin independent precocious puberty. J Pediatr Endocrinol Metab.

[11] Frisch LS, Copeland KC, Boepple PA (1992). Recurrent ovarian cysts in childhood: diagnosis of McCune-Albright syndrome by bone scan. Pediatrics.

[12] Kdous M, Hachica R, Gamoudi A (2004). Early isosexual precocious pseudopuberty revealing a juvenile granulosa cell tumor in a six-year-old girl. Gynecol Obstet Fertile.

[13] Barber HRK, Graber EA (1973). Gynecological tumors in childhood and adolescence. Obstet Gynecol Surv.

[14] Adelman S, Benson CD, Hertzler JH (1975). Surgical lesions of the ovary in infancy and childhood. Surg Gynecol Obstet.

[15] Carson MJ (1953). Clinical conference at the Los Angeles Childrens Hospital: Case 3.Ovarian feminizing tumors. J Pediatr.

[16] Faber HK (1962). Meigs’ syndrome with thecomas of both ovaries in a 4-year-old girl. J Pediatr.

[17] Fink D, Kubik-Huch RA, Wildermuth S (2001). Juvenile granulosa cell tumor. Abdom Imaging.

[18] Raafat F, Klys H, Rylance G (1990). Juvenile granulosa cell tumor. Fetal Pediatr Pathol.

[19] Raney RB, Sinclair L, Uri A, Schnaufer L, Cooper A, Littman P (1987). Malignant ovarian tumors in children and adolescents. Cancer.

